# Long‐term remission of extramedullary cutaneous relapse of acute myeloid leukaemia (leukaemia cutis) treated with decitabine‐venetoclax

**DOI:** 10.1002/jha2.388

**Published:** 2022-01-28

**Authors:** Denise Maravalle, Alessandra Filosa, Catia Bigazzi, Guido Collina, Piero Galieni

**Affiliations:** ^1^ Department of Haematology and Stem Cell Transplantation Unit C. e G. Mazzoni Hospital Ascoli Piceno Italy; ^2^ Department of Anatomical Pathology C. e G. Mazzoni Hospital Ascoli Piceno Italy

**Keywords:** AML, cyclic therapy, decitabine, leukaemia cutis, long‐term remission, venetoclax

## Abstract

In February 2020, a 74‐year‐old female was diagnosed with myelomonocytic acute myeloid leukaemia with FLT3 mutation and blasts positive for CD33, BCL‐2 and CD68/PGM1. Not responding to a standard Cytarabine–containing regimen plus Midostaurin, the patient achieved a complete remission (CR) of the disease in the bone marrow following a reinduction therapy with high‐dose Cytarabine but simultaneously relapsed developing leukaemia cutis with disseminated lesions in 80% of the body surface area. After receiving 10 cycles of Decitabine plus Venetoclax the patient achieved and maintains a continuous CR.

## 1 INTRODUCTION

Extramedullary (EM) involvement in patients with acute myeloid leukaemia (AML) can present in various tissues with or without systemic disease. Leukemic cells can infiltrate any site of the body, most often soft tissue, but skin, bones, central nervous system and lymphatic system may also be affected [1]. An analysis of AML with EM disease detected the overrepresentation of abnormalities like monocytic differentiation and CD56 expression, as well as cytogenetic results of inversion (16)(p13.1q22) and translocation (8;21)(q22;q22.1) [2]. Although relatively rare, the EM manifestation of AML is clinically significant, as its therapeutic treatment becomes a problem that is difficult to solve: in fact, treatment approaches to EM disease mainly depend on whether the infiltration of EM organs by leukemic cells presents at initial diagnosis or relapse, and even the site of relapse plays a determinant role. The infiltration of the skin by leukemic cells, called leukaemia cutis (LC), occurs in about 3% of patients with AML and is frequently associated with monocytic and myelomonocytic differentiation; though affecting only the skin, with localized or disseminated lesions, LC almost always represents a cutaneous manifestation of an underlying bone marrow disease and therefore systemic treatment should be administered [1]. A skin biopsy is necessary for establishing the correct diagnosis, and in patients presenting with a single skin lesion with symptoms of mass effect surgical intervention may be indicated before the systemic chemotherapy [3]. Until recently, regardless of bone marrow involvement, a Cytarabine–containing regimen has been the most reasonable approach to LC [4]. Targeted agents like BCL‐2 inhibitors (Venetoclax), hypomethylating agents (Decitabine and 5‐Azacitidine) and Gemtuzumab Ozogamicin have also been used successfully. In addition, eligible patients with or without bone marrow disease can receive hematopoietic stem‐cell transplantation (HSCT) [3]. Here we report a case of LC relapse, without bone marrow involvement, in a patient with AML who achieved and maintains a continuous complete remission (CR) with a long cyclic administration of Decitabine and Venetoclax (VEN‐DEC).

## 2 CASE DESCRIPTION

**FIGURE 1:**
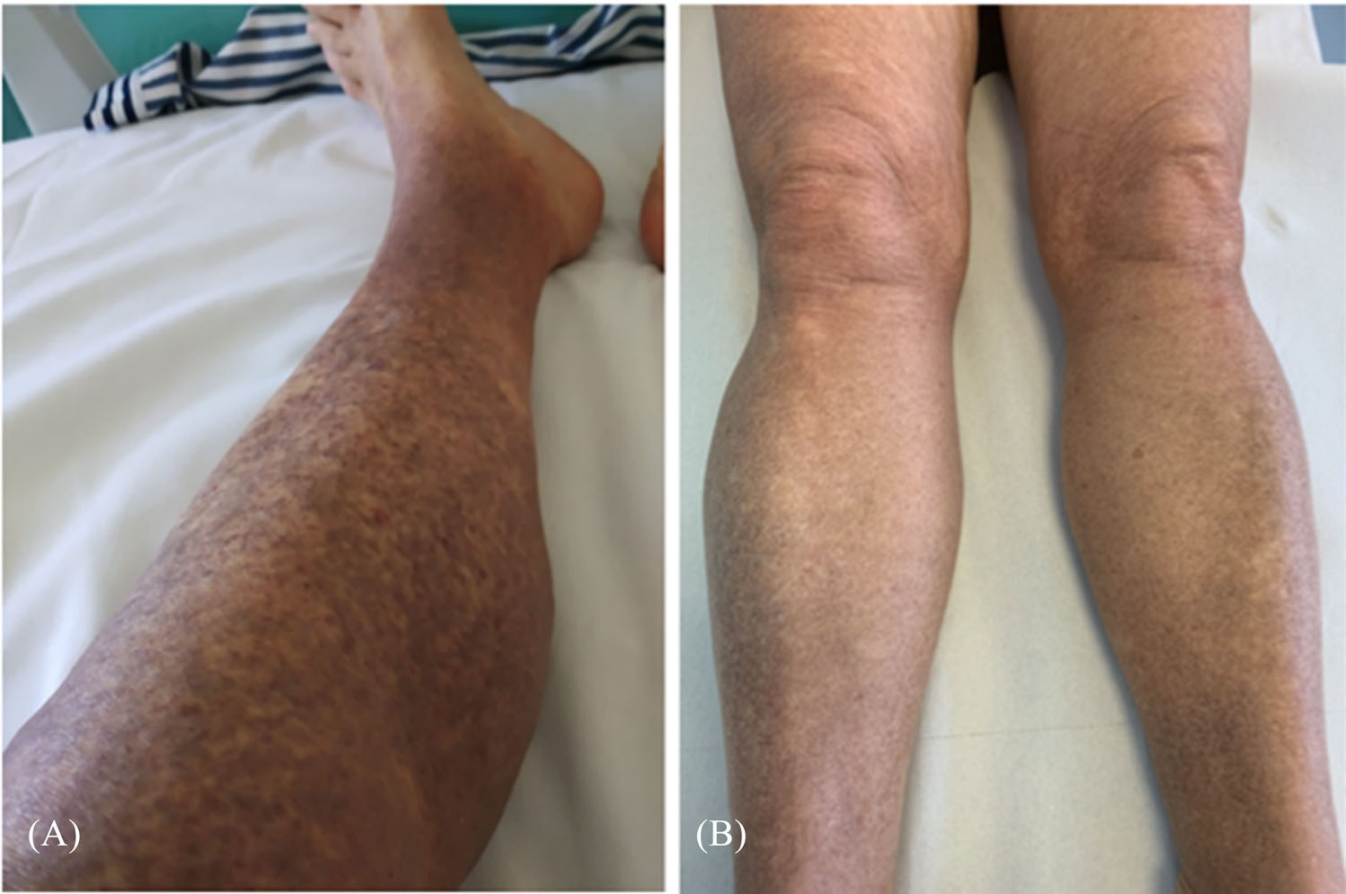
Clinical manifestation of leukaemia cutis. (A) Maculopapular rash appeared on the lower limbs before starting therapy with decitabine‐venetoclax (VEN‐DEC). (B) Complete morphological resolution of the skin lesions after the second course of VEN‐DEC therapy

**FIGURE 2:**
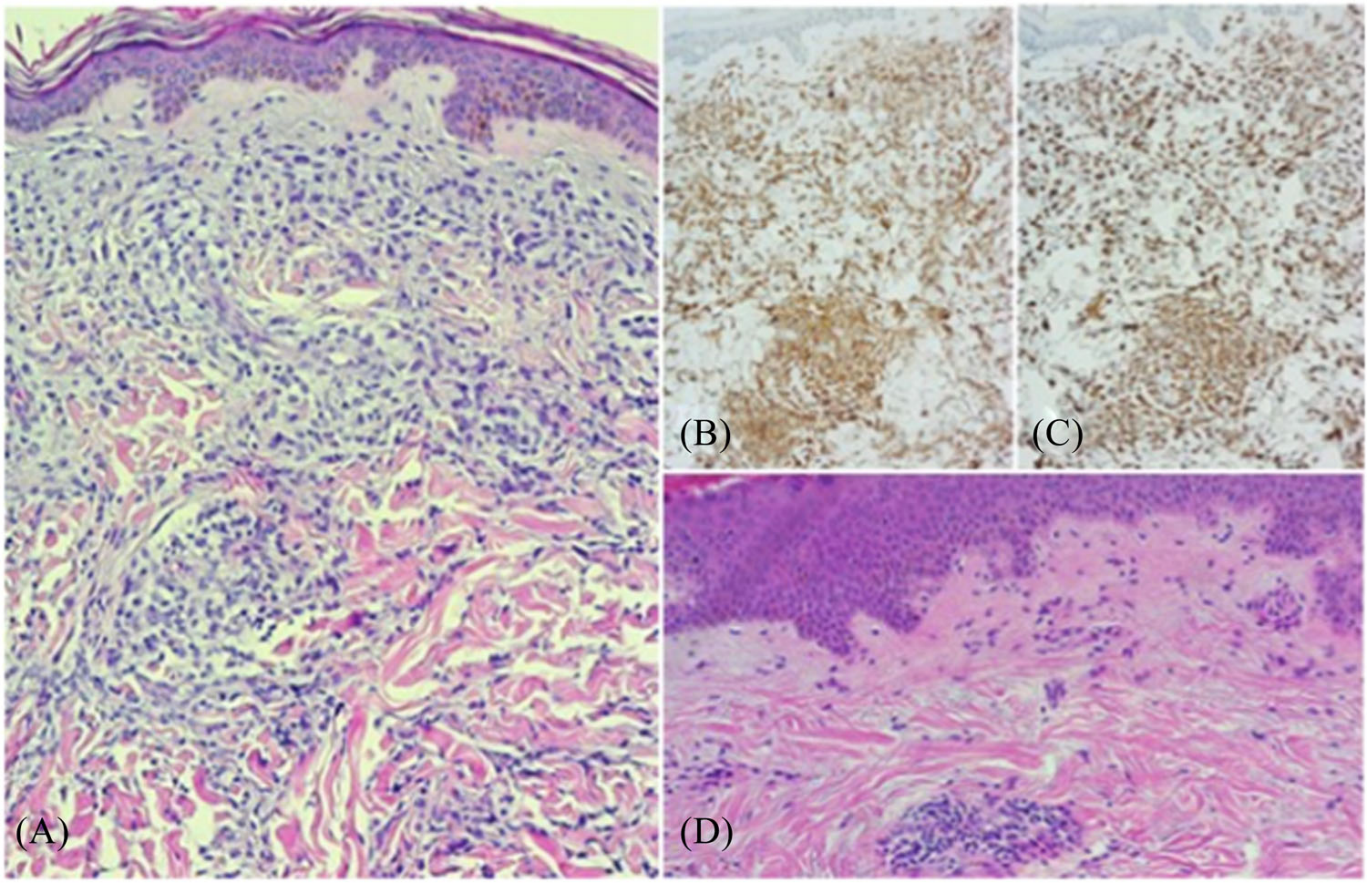
Histological appearance of leukaemia cutis. (A) Pre‐therapy skin biopsy and morphological examination: perivascular and interstitial infiltrate of myeloid blasts in the papillary and reticular dermis (H&E 40x). (B) Immunohistochemical results: diffuse CD33 staining in myeloid blasts. (C) Immunohistochemical results: intense diffuse BCL‐2 immunostaining in myeloid blasts. (D) Post‐therapy skin biopsy and morphological examination: normal epidermal and dermal outline with a mild and focal perivascular infiltrate of small lymphocytes

A 74‐year‐old female with hyperleukocytosis and thrombocytopenia presenting with dizziness, fatigue after physical activities, night sweats and fever, was admitted to our hospital in February 2020. In 2011, the patient, diagnosed with breast cancer, had undergone radical left breast removal and had been treated with radiotherapy and hormone therapy. Laboratory tests indicated the following values: WBC 14,000/mmc with circulating monocytoid blast cells, normal levels of haemoglobin and low platelet count. A bone marrow aspirate confirmed the diagnosis of myelomonocytic AML, and bone marrow biopsy revealed diffuse infiltration by blasts (90% of the total) with both a myeloid and monocytic immunophenotype positive for CD33, BCL‐2 and CD68/PGM1 (about 10%). Blasts were only occasionally immunoreactive for CD34, CD117 and myeloperoxidase (MPO). Flow cytometric analysis of bone marrow aspirate showed blast cells CD64+CD33+CD14‐CD35‐, cytogenetic analysis of leukemic cells showed a normal 46,XX karyotype, NGS molecular analysis found FLT3‐TKD, NPM1A, IDH2, DNMT3A mutations, and overexpressed WT1. Mild gingival hyperplasia was evident; there were no signs of the cutaneous manifestation of the disease. The patient was treated with Daunorubicin 60 mg/mq on days 1–3, Cytarabine 100 mg/mq by continuous intravenous infusion on days 1–7 and Midostaurin 50 mg every 12 h on days 8–21, without achieving a CR. The patient underwent a second chemotherapy course with high‐dose Cytarabine (1.5 g/mq twice a day) on days 1‐3‐5 followed by Midostaurin at the same dosage as previous therapy, and CR was achieved. Simultaneously with haematological recovering after reinduction therapy, a maculopapular rash appeared on the lower limbs, rapidly spreading to about 80% of the body surface area (Figure 1A). Histological examination of biopsy samples of the skin revealed a perivascular and periadnexal leukemic infiltrate with a clear‐cut monocytic cell morphology and immunophenotype. Blasts were diffusely positive for CD33, CD68/PGM1 and BCL‐2 (Figure 2A–C) and negative for both CD34 and MPO. The patient underwent a new bone marrow biopsy and bone marrow aspirate with immunophenotypic evaluation, which confirmed CR. Given the proven effectiveness of BCL‐2 inhibitor in combination with hypomethylating agents in AML, including EM involvement [5, 6, 7], and the intense diffuse positivity for BCL‐2, the patient was treated with Decitabine 20 mg/mq/day for 5 days plus Venetoclax at the dosage of 400 mg/day, together with antibacterial and antifungal oral prophylaxis. The first course of therapy was interrupted after 21 days of Venetoclax administration due to severe and persistent pancytopenia, detected after seven days of treatment. Granulocyte growth factor filgrastim was given daily from day +7 until haematological recovery, which was achieved 2 weeks after interruption of treatment. The result after the first cycle of VEN‐DEC therapy was very satisfactory, with a clearly evident reduction in cutaneous manifestations. Complete resolution of the skin lesions was achieved after the second course of therapy (Figure 1B). In total, 10 cycles of VEN‐DEC therapy were administered on an outpatient basis, except for the first one; each cycle started 6 weeks after the previous one, due to neutropenia, with reduced dose of Venetoclax from the third cycle as follow: Decitabine 20 mg/mq/day for 5 days in combination with Venetoclax at the dosage of 400 mg/day for 14 days. During each cycle filgrastim was always administered from day +7, together with antibacterial and antifungal oral prophylaxis, until neutrophil recovery. A febrile episode, occurred during the first course of therapy, was managed by intravenous broad‐spectrum antibiotics. The patient received a total of 10 red blood cell units and three platelet apheresis, all of them in the first three courses of therapy. Medullary evaluations were carried out after the second, fourth, and eighth cycle of VEN‐DEC therapy with evidence of continuous CR, confirmed by immunophenotypic evaluation. A skin biopsy was performed after the fourth and tenth cycle of treatment and showed the absence of blast cells (Figure 2D). Therapy was discontinued in 28 May 2021 and to date (04 October 2021) the patient is still in continuous CR.

## 3 DISCUSSION

In this case the cutaneous manifestation of AML, recently diagnosed and without any signs of skin involvement at its onset, was unusual and unexpected since it occurred during the administration of systemic chemotherapy, and after having just achieved the CR of the disease in the bone marrow. The morphological and immunohistochemical differences between bone marrow blasts at diagnosis and skin blasts after systemic chemotherapy, with loss of positivity for MPO and CD34, may explain the change in the disease and its marked skin tropism. In fact, LC is almost always associated with monocytic differentiation, and staining for MPO may be absent [8], The manifestation of LC has been associated with more aggressive disease, poor outcomes, and shorter survival because patients have poor response rates with conventional chemotherapy. AML‐type regimens are frequently recommended as initial therapy, as reported in the National Comprehensive Cancer Network and European Leukemia Net guidelines (2017). In this case, the patient had already been treated at the onset of disease using standard Cytarabine–containing regimens with the addition of FLT3 inhibitor Midostaurin, and yet complete response in the bone marrow and cutaneous manifestation of AML occurred simultaneously. Patients who relapse with LC without leukaemia in the bone marrow are considered for total skin electron beam (TSEB) therapy since low‐dose is well tolerated with minimal toxicity, but a series reviewing TSEB outcomes had reported that while 50% of patients achieved a complete response to therapy, 1‐year local control was only 33% [9]. Successful treatment of LC is crucial in preventing skin blasts from reseeding the bone marrow, thus we thought about alternative regimens. The use of Venetoclax, a potent and selective oral BCL‐2 inhibitor, has shown great efficacy in the treatment of AML in combination with hypomethylating agents [8, 10, 11]. However, few data exist about the effectiveness of this drug on EM disease, and specifically on LC [5, 7]. Furthermore, the dosage, modality and duration of treatment have not been established yet. In February 2020 the Italian medicines agency AIFA, after FDA and EMA, approved the use of Venetoclax in combination with Azacitidine or Decitabine in patients with newly diagnosed AML aged 75 years and over or with comorbidities that preclude the administration of intensive chemotherapy. For the treatment of our AML patient with skin relapse without bone marrow involvement, Venetoclax and Decitabine were used off‐label. This therapy resulted in a quick complete response; it was well tolerated, with an acceptable safety profile, and without unusual haematological toxicities. With carefully planned cycles of treatment over an extended period of time, the patient obtained a continuous CR of disease and excellent quality of life. Time will tell whether VEN‐DEC cyclic therapy will translate into the treatment protocol for LC.

## 4 CONCLUSION

LC is a rare manifestation of AML and gives the disease a worse prognosis. In our clinical case, the use of Venetoclax combined with hypomethylating agent Decitabine showed good results: VEN‐DEC might become a good therapeutic option in the management of patients with EM AML, with the aim to perform allogeneic HSCT with the deepest response. Based on our clinical case experience, we suggest that patients ineligible for allogeneic HSCT continue the cycles of treatment with VEN‐DEC for as long as clinically indicated and, in the short term, avoid any interruptions of therapy.

## CONFLICT OF INTEREST

The authors declare that they have no conflict of interest.

## AUTHOR CONTRIBUTIONS

Denise Maravalle, Catia Bigazzi and Piero Galieni diagnosed and treated the patient, provided the clinical samples and wrote the paper. Alessandra Filosa and Guido Collina performed histological and morphological analyses of patient biopsies. All authors analyzed the data, reviewed the paper and approved the final version of the manuscript.
